# The Book World of Medicine and Science

**Published:** 1901-06-22

**Authors:** 


### The Book World of Medicine and Science.

Burdett's Hospitals and Charities 1901, being the Year-Book of Philanthropy and the Hospital Annual. By Sir Henry Burdett, K.C.B. (London: The Scientific Press. 1901. Price 5s.)

Concerning the directory portion of this admirable book we have no comment to make beyond saying that it maintains its old reputation, giving as it does a full and up-to-date record of the medical colleges, the hospitals, dispensaries, poor law infirmaries, lunatic asylums, convalescent homes, nursing institutions, military and naval hospitals, and a crowd of other medical and philanthropic institutions in the United Kingdom, besides dealing with the hospitals in the Colonies and the United States. In regard to all these the information given is curiously full and complete, and is arranged in much the same way as in former years. The first part of the book contains a series of freshly written chapters on various subjects of more or less importance to those interested in hospitals and charities. It might be thought that all had been said that could be said on these subjects, but every year Sir Henry Burdett seems to be able to lay hold of some point or other which requires consideration.

Among other matters dealt with in this volume is the ever-growing cost of the nursing department in our great hospitals. Sir Henry Burdett points out that this is due to the great change that has taken place of recent years in the uses to which a hospital is put. Formerly a large number of cases of a chronic character were met with among the in-patients; now the inmates are suffering for the most part from acute diseases, and as the great aim is "to cure the greatest number of patients in the fewest possible beds in the shortest possible time," every case requires individual attention, and the cost runs up. Still there ought to be a limit to this form of expenditure, and the question arises whether we have not reached, and in some cases overpassed, this line. "Nowadays," says Sir Henry Burdett, "the danger is a growing tendency on the part of some in authority to unduly increase the numbers of the nursing staff, and to add to the expenditure upon this one item sums which can and ought to be saved. There is a unit of efficiency which can be attained for a maximum sum per head per annum, which unit ought to be ascertained and strictly adhered to in every well-managed hospital." Some idea of the increase in the cost of nursing at certain hospitals may be gathered from the statement that at the London Hospital, while in the year 1878 there was one nurse to every four beds occupied, there is now nearly one nurse for every two beds, and that at Guy's, while there used to be one nurse to every four beds, there was in 1898 one nurse to less than two beds. It has also to be remembered, in considering the meaning of this increase in the number of nurses and its relation to expenditure, that "the modern nurse necessitates the employment of a troop of ward-maids, entailing a large expenditure, which makes the growth in nursing expenditure something like 200 per cent. compared with what it was five-and-twenty years ago." Sir Henry Burdett maintains that what we want settled is an average maximum sum per occupied bed as the recognised unit of expenditure upon the nursing department, and that this should be adopted by every well-regulated hospital so as to prevent any further large increase in the sum devoted to nursing in hospitals in this country. We would point out, however, that although it may be a perfectly legitimate aim to arrive at such an average maximum, which should not be exceeded—taking, of course, the average of many hospitals or of the several wards within the walls of one great hospital—the very word "average" involves the fact that some must be above and some below, and thus that it will never be possible to judge of the propriety of the nursing arrangements of a given hospital by reference merely to the proportion between nurses and patients, or between nursing and general expenditure, valuable as such a record will be as suggesting inquiry in cases in which the average may be exceeded. The question of ward-maids is also considered, and it is held that in practice it would be "more economical and satisfactory to provide accommodation for an adequate number of resident ward-maids than to depend upon the casual labour of scrubbers which may be obtained from outside," a statement which, of course, touches upon the burning question of living "in" or "out." Probably it will turn out that in different localities different solutions will have to be given to the problem. Many other topics are touched upon, and on the whole the first half of the book will be found as suggestive to the reader as the second is essential to those who require accurate information regarding hospitals and charities.

The Microscopy of the Starches. By Hugh Galt M.B., C.M. (London: Baillière, Tindall and Cox. 1900. Illustrated. Pp. 104. Price 3s. 6d.)

Dr. Galt, the Professor of Forensic Medicine at Glasgow, embodies in this brief monograph the results of unremitting attention to a subject that has been singularly overlooked. There is no doubt that the author's hopes will be fulfilled, and that his work may prove of value to "laboratory workers, analysts, brewers and bakers." We would say "and to clinicians also," for we have a very lively recollection of the whole of the resident staff, and many of the visiting staff of a famous London hospital, being puzzled by the appearance in the saliva of a man home from the tropics of certain oval bodies. The idea became current that these bodies were the ova of some extraordinary and little known parasite; but it was reserved for the greatest living authority on tropical diseases to discover that these "bodies" were "starch grains." Dr. Galt describes the microscopic characters of each of some fifteen varieties of starch, and illustrates his descriptions by very beautiful micro-photographs of very great precision and value. We trust that Dr. Galt will continue his researches, for they cannot fail to prove of great importance, especially to those engaged in commercial matters and in the detection of adulteration.

